# Chronic behavioral and seizure outcomes following experimental traumatic brain injury and comorbid *Klebsiella pneumoniae* lung infection in mice

**DOI:** 10.1111/epi.18551

**Published:** 2025-07-15

**Authors:** Sarah S. J. Rewell, Ali Shad, Lingjun Chen, Erskine Chu, Jiping Wang, Ke Chen, Terence J. O'Brien, Jian Li, Pablo M. Casillas Espinosa, Bridgette D. Semple

**Affiliations:** ^1^ Department of Neuroscience The School of Translational Medicine, Monash University Melbourne Victoria Australia; ^2^ Alfred Health Melbourne Victoria Australia; ^3^ Department of Microbiology Monash Biomedical Discovery Institute, Monash University Clayton Victoria Australia; ^4^ Department of Medicine (Royal Melbourne Hospital) University of Melbourne Parkville Victoria Australia

**Keywords:** bacteria, behavior, controlled cortical impact, epilepsy, hospital‐acquired infection, pneumonia, seizures

## Abstract

**Objective:**

Traumatic brain injury (TBI) is a leading cause of long‐term disability, and infections such as pneumonia represent a common and serious complication for patients with TBI in the acute and subacute post‐injury period. Although the acute effects of infections have been documented, their long‐term consequences on neurological and behavioral recovery as well as the potential precipitation of seizures after TBI remain unclear. This study aimed to investigate the chronic effects of *Klebsiella pneumoniae* infection following TBI, focusing on post‐traumatic seizure development and neurobehavioral changes.

**Methods:**

Using a mouse model, we assessed the long‐term effects of TBI and *K. pneumoniae* infection both in isolation and in combination.

**Results:**

We found that, although infection with *K. pneumoniae* resulted in loss of body weight and increased mortality compared to vehicle‐inoculated mice, there was no additional mortality in TBI animals. Furthermore, although TBI alone induced chronic hyperactivity and reduced anxiety‐like behaviors, *K. pneumoniae* lung infection had no lasting effect on these long‐term outcomes. Third, although TBI resulted in both spontaneous and evoked seizures long‐term post‐injury, early post‐injury *K. pneumoniae* infection did not affect late‐onset seizure susceptibility.

**Significance:**

Together with recent findings on acute outcomes in this combined insult model of TBI and *K. pneumoniae* infection, this study suggests that *K. pneumoniae* does not significantly alter long‐term neurobehavioral outcomes or the development of post‐traumatic epilepsy. This research highlights the need to further explore the interplay between additional immune insults such as infection that may influence long‐term recovery.

**Key points**

- A mouse model of traumatic brain injury (TBI) and *Klebsiella pneumoniae* lung infection has been established.
- Six months post‐injury/infection, TBI induced chronic behavioral deficits and seizure susceptibility.
- The additional insult of a *K. pneumoniae* lung infection did not exacerbate chronic outcomes.

## 1 BACKGROUND

Traumatic brain injury (TBI) is a leading cause of long‐term disability worldwide, with many survivors experiencing persistent cognitive, emotional, and physical impairments. Among the most significant acute and subacute complications following severe TBI are hospital‐acquired infections, particularly pneumonia, which affects a substantial proportion of patients.^1^, ^2^, ^3^, ^4^ Pneumonia caused by *Klebsiella pneumoniae*, an opportunistic pathogen commonly associated with ventilator‐associated infections, is of particular concern due to its role in exacerbating systemic inflammation and respiratory failure, and increasing the risk of mortality.^4^, ^5^, ^6^ In patients with TBI, immune dysregulation induced by the injury itself heightens vulnerability to such infections, which can worsen functional outcomes and complicate recovery.^7^, ^8^, ^9^ Several recent studies, including from our group, have investigated the acute consequences of various infection models in the context of TBI and provided important insights into how bacterial infections exacerbate acute post‐injury neuroimmune responses.^10^, ^11^, ^12^, ^13^, ^14^ However, there remains a critical gap in understanding how pneumonia following TBI impacts long‐term neurological and behavioral outcomes.

Post‐traumatic epilepsy (PTE) represents a debilitating long‐term complication for survivors of moderate or severe TBI.^15^, ^16^, ^17^, ^18^, ^19^, ^20^ Defined as recurrent and unprovoked seizures that occur at least 1 week after TBI,^21^ PTE can severely impact quality of life and is associated with an increased risk of cognitive decline, mood disorders, early‐onset neurodegeneration, and mortality.^22^, ^23^, ^24^, ^25^, ^26^ Yet the precise mechanisms that drive the development of epileptogenesis after a brain injury remain elusive. Neuroinflammation, a well‐known hallmark of TBI, is thought to play a role, and in this way, an additional immune challenge such as an infection is hypothesized to promote post‐traumatic epileptogenesis.^27^, ^28^

To address this hypothesis, we recently conducted a retrospective cohort study examining a large trauma registry of adults with moderate to severe TBI. Infections were documented for approximately one quarter of TBI patients in the registry, with pneumonia being the most common presentation. By multivariate analysis to adjust for known risk factors, we found a solid association between hospital‐acquired infections and the development of PTE at 2 years post‐injury.^29^ This finding suggests that hospital‐acquired infections may contribute to the development of PTE, such that infections represent a modifiable risk factor. However, further exploration of this hypothesis in mouse models have to date failed to produce experimental evidence to support this theory. Specifically, we have evaluated the long‐term consequences of peripherally‐administered lipopolysaccharide (LPS), as an infection‐like immune challenge, after experimental TBI in mice. In both pediatric and adult contexts, we reported that LPS induced a robust acute immune response yet did not exacerbate the long‐term development of post‐traumatic seizures.^10^, ^30^

Although the LPS mouse model has some advantages as a well‐established, predictable model of a systemic immune challenge, it fails to recapitulate many of the key features of a live bacterial infection in vivo. As such, it is pertinent that experimental models shift toward preferential use of live infectious agents, delivered via clinically‐relevant routes, to more appropriately model the complex pathophysiological scenario of a hospital‐acquired infection in an individual with severe TBI.^14^ To address this, we recently established a new model of intratracheal inoculation of *K*. *pneumoniae* bacteria after experimental TBI in the mouse, and conducted detailed characterization of the acute consequences of this dual insult.^11^ We observed that *K. pneumoniae* lung infection after TBI induced a robust, yet transient, inflammatory response, restricted primarily to the lungs but with some systemic effects, alongside exacerbated elevation of several pro‐inflammatory genes such as *Ccl2* in the brain of TBI+ *K. pneumoniae* mice. However, the potential long‐term consequences of these changes were not determined.

The current study, therefore, sought to address this knowledge gap by investigating the chronic consequences of *K. pneumoniae* infection following experimental TBI, with a particular focus on the development of post‐traumatic seizures and alterations in neurobehavioral outcomes.

## 2 METHODS

### 2.1 Experimental timeline

**FIGURE 1:**
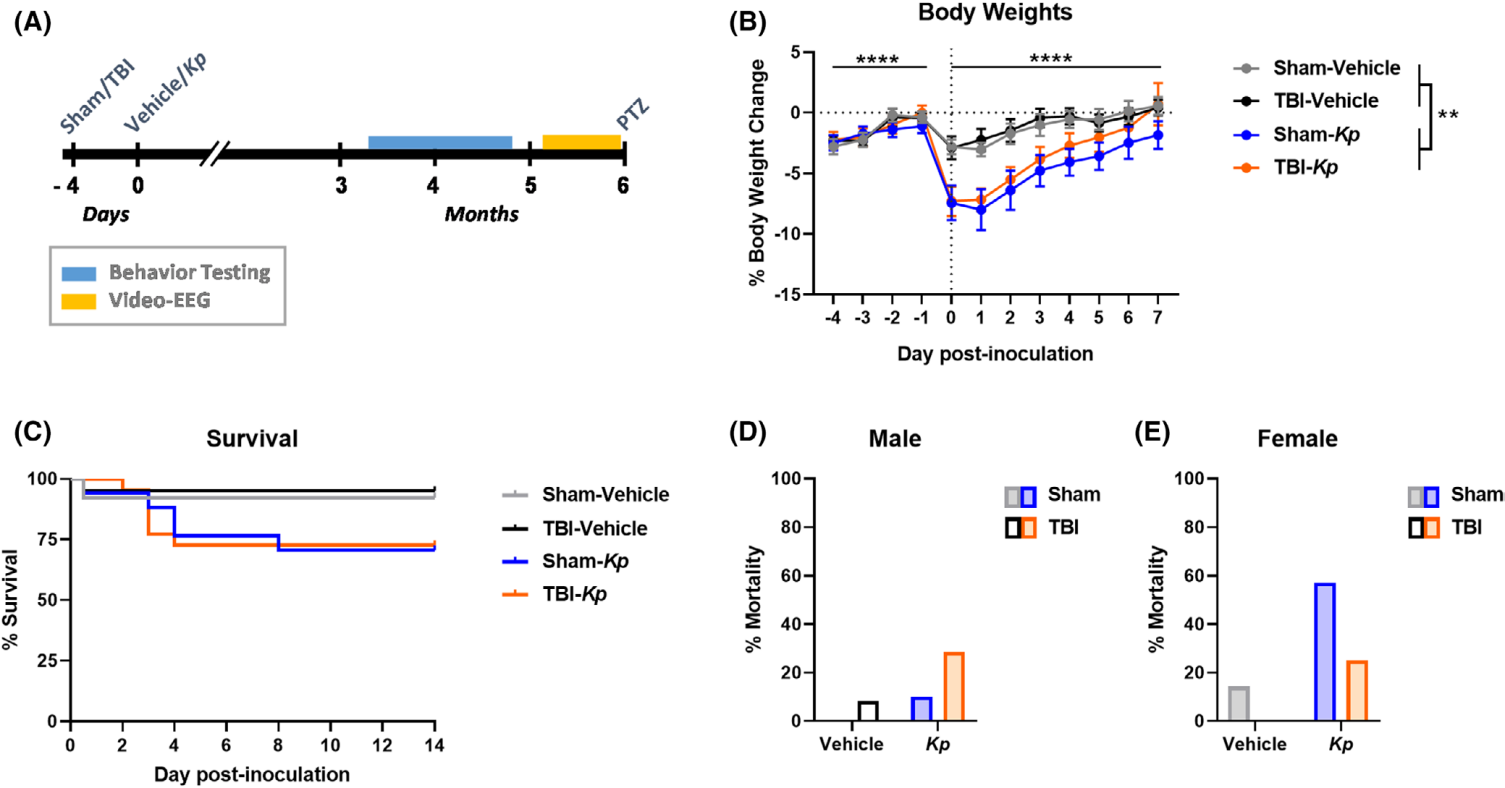
Experimental timeline and acute outcomes. (A) Experimental timeline. (B) Body weight changes from the time of TBI (traumatic brain injury) or sham procedure (Day −4) then up to 7 days post‐inoculation with *Kp* (*Klebsiella pneumoniae*) or Vehicle. **p < 0.01 indicates main effect of *Kp* from a three‐way ANOVA. (C) Percent survival over the first 2 weeks post‐inoculation. (D) Mortality rate in males. (E) Mortality rate in females.

To determine the long‐term effects of lung infection with a TBI, mice were subjected to a moderate‐to‐severe TBI model (or a sham control surgery), followed by intratracheal inoculation with vehicle or *K. pneumoniae* bacterium at 4 days post‐injury. The four experimental groups were Sham‐Vehicle, Sham‐*Kp*, TBI‐Vehicle, and TBI‐*Kp*. At ~4 months (16 weeks) post‐injury, mice underwent extensive behavioral testing over a 3‐week period. At ~4–5 months post‐injury, a recording electrode was implanted to allow for subsequent video‐EEG (electroencephalography) monitoring for an average of 10 days per mouse. Finally, all mice received a sub‐convulsive dose of pentylenetetrazol (PTZ) to evaluate evoked seizure responses, followed by tissue collection at 6 months post‐injury (Figure 1A).

### 2.2 Animals and ethics

All animal experiments were conducted with approval from the Alfred Research Alliance Animal Ethics Committee (#P8032) and the Animal Care and Use Review Office (ACURO) of the U.S. Department of Defense. These procedures adhered to the approved standards and the Australian Code for the Care and Use of Laboratory Animals as set by the National Health and Medical Research Council of Australia (NHMRC). Male and female C57Bl/6J mice were obtained from the Walter and Eliza Hall Institute of Medical Research in Melbourne, Australia, and acclimated for 1 week before experiments began. (See Supporting Information for further detail.)

### 2.3 Controlled Cortical Impact (CCI) model of TBI

Moderate‐to‐severe experimental TBI was induced in 10‐ to 12‐week‐old mice using the Controlled Cortical Impact (CCI) model, as described previously^30^ (see Supporting Information). Sham animals underwent the same surgical procedure without the impact.

### 2.4 *K. pneumoniae* lung infection model

Freeze‐dried cultures of *K. pneumoniae* ATCC 15380 were obtained from In Vitro Technologies (Noble Park, VIC, Australia) and cultured on nutrient agar plates (Medium Preparation Unit, University of Melbourne, Victoria, Australia) at 37°C overnight. For the initial culture, a large number of *K. pneumoniae* colonies were harvested and suspended in cation‐adjusted Mueller‐Hinton broth (CAMHB) for an additional 24‐h incubation at 37°C with shaking at 180 rpm. Mid‐logarithmic‐phase cultures were then prepared by incubating in fresh CAMHB for 3 h. The bacterial concentration (colony‐forming units [CFU]/mL) was assessed by measuring the optical density (OD) at 600 nm and adjusted to the desired working concentration.

Mice were randomized for inoculation with 10^6^ CFU *K. pneumoniae* or vehicle on Day 4 post‐injury, as hospital‐acquired infections are most common during the first week after a TBI.^31^, ^32^ A series of pilot studies were conducted previously to determine the optimal dose of *K. pneumoniae* via intratracheal inoculation to achieve an appropriate lung infection model in adult male and female mice.^11^ For inoculation, mice were anesthetized with 2%–4% inhaled isoflurane by an experienced technician, and a MicroSprayer Aerosolizer (Penn‐Century, Philadelphia, PA, USA) was used to administer 25 μL bacterial suspension or vehicle solution (diluted CAMHB) directly into the trachea.^33^ Five‐fold serial dilutions of both *K. pneumoniae* and vehicle samples were spiral plated and incubated overnight at 37°C on nutrient agar plates to verify the CFU/mL in the administered inoculum, using ProtoCOL 3 software (Synbiosis, USA). Following recovery from the procedure, mice were closely monitored post‐treatment for sickness behavior, general appearance, and weight loss over the time course. Weight loss ≥20% was a trigger for immediate humane euthanasia. Animal numbers are depicted in Table 1.

**TABLE 1:** Experimental animal numbers.

	Sham‐Vehicle	Sham‐*Kp*	TBI‐Vehicle	TBI‐*Kp*
Male	6 (0)^a^	9 (1)	11 (1)	10 (4)
Female	6 (1)	3 (4)	8 (0)	6 (2)
Total	12 (1)	12 (5)	19 (1)	16 (6)

^a^ Numbers in table refer to experimental animals that reached the original experimental end point, whereas those in parentheses reflect those that were euthanised due to excessive weight loss during the experimental period, in line with animal ethics guidelines. *Kp* = *Klebsiella pneumoniae*; TBI = traumatic brain injury.

### 2.5 Behavior testing

At ~16 weeks post‐injury, a comprehensive battery of neurobehavioral tests was conducted to assess the chronic consequences of TBI and *K. pneumoniae*. Tests included the Open Field test, Elevated Plus Maze, Rotarod, Three‐Chamber Social Approach test, and Sucrose Preference test, as detailed in the Supporting Information.

### 2.6 Video‐electroencephalography (EEG)

To evaluate chronic seizure activity, EEG electrodes were surgically implanted at 18–19 weeks post‐injury. Under isoflurane anesthesia, epidural recording electrodes (E363/20/2.4/SPC ELEC W/SCREW SS, Plastics One Inc., USA) were carefully positioned: one ipsilateral and distal to the craniotomy, one contralateral to the craniotomy (2.5 mm to the right of the midline, −2.5 mm relative to Bregma), and two electrodes over the cerebellum for ground and reference.^34^ An additional anchor screw (00‐96 × 3/32, Plastics One Inc.) was placed over the left frontal region to reinforce the head cap. All screws were secured to the skull with SuperGlue (Bostik, Australia), and the electrodes were mounted into a pedestal head cap (MS363, Plastics One Inc.) and fixed with dental acrylate. The skin was sutured around the head cap, and the animals received subcutaneous pain relief (buprenorphine 0.05 mg/kg and bupivacaine 1 mg/kg) along with saline for hydration. Following electrode implantation, mice were housed individually for the remainder of the experiment.

### 2.7 Phenotyping post‐traumatic epilepsy

From 5 months post‐TBI, continuous video‐EEG recordings were collected using a Grael EEG amplifier (Compumedics, Australia), accompanied by infrared video recordings. The EEG data were collected with a high‐pass filter at 1 Hz and a low‐pass filter at 70 Hz, at a sampling rate of 512 Hz, and digitized using Compumedics Profusion EEG software v 4.0. Recordings were obtained from both ipsilateral and contralateral electrodes, using common ground and reference signals. Each animal had a total of 8–12 days of video‐EEG data recorded, which was analyzed using Assyst.^35^ Seizures were identified by changes in the EEG pattern lasting more than 10 s, with an amplitude greater than three times the baseline, a repetitive and rhythmic discharge pattern, and variations in amplitude at the start and end of the seizure.^30^ Potential seizure events identified by Assyst were reviewed by an experienced investigator (P.M.C.E.), who examined the EEG tracing blinded to the experimental group. Finally, identified seizures were confirmed following review of the video recording. Approximately 20% of video‐EEG recordings were of insufficient quality to allow for accurate quantification, and were excluded. The presented results are from the remaining 80% of recordings.

### 2.8 Pentylenetetrazol seizure susceptibility challenge

Immediately before tissue collection at 24 weeks post‐TBI, a single intraperitoneal dose of 40 mg/kg PTZ (P6500, Sigma, Australia) was administered to assess susceptibility to evoked seizures as an additional, indirect measure of PTE development. Behavioral responses to PTZ were observed over a 15‐min period. An experienced investigator (S.S.R.), blinded to experimental group, reviewed the video recordings and rated the response to PTZ according to a modified 7‐point Seizure Severity Score, where 0 indicates no response or normal activity, and 7 indicates status epilepticus leading to death.^30^, ^36^, ^37^

### 2.9 Statistical analysis

Statistical analysis was performed using GraphPad Prism v.9.4.1 (GraphPad Software Inc., San Diego, CA, USA), with significance defined as *p* < 0.05. Two‐ and three‐way analyses of variance (ANOVAs) were performed with Tukey's post hoc test where appropriate. Data with three independent variables of time, injury, and infection, were assessed with a three‐way ANOVA. In most instances, both male and female mice were pooled per group, with open circle data points graphically denoting female animals. Potential sex differences were tested by three‐way ANOVA (factors of sex, injury, and infection) where appropriate, and reported only where significant sex differences were detected. Differences in mortality were tested using the Log‐Rank (Mantel−Cox) test. Data are presented as mean ± standard error of the mean (SEM).

## 3 RESULTS

### 3.1 Impact of *K. Pneumoniae* infection on body weight and mortality in TBI mice

We sought to test the hypothesis that lung infection with *K. pneumoniae* after a moderate‐to‐severe experimental TBI would exacerbate chronic behavioral and seizure outcomes. To determine this, four experimental groups (Sham‐Vehicle, Sham‐*Kp*, TBI‐Vehicle, and TBI‐*Kp*) were compared across a range of outcome measures (Figure 1A).

Body weights were monitored as an indicator of general health. When compared across the first week post‐infection, a reduction in body weight due to *K. pneumoniae* inoculation is evident in Sham‐*Kp* and TBI‐*Kp* groups (Figure 1B) compared to vehicle‐treated groups. Three‐way ANOVA confirmed a main effect of time (*F*_4,222_ = 28.36, *p* < 0.0001), a main effect of *Kp* (*F*_1,57_ = 9.36, *p* = 0.0034), and a significant time × *Kp* interaction (*F*_1,57_ = 9.06, *p* < 0.0001). However, no effect of TBI alone was observed (*F*_1,57_ = 0.51, *p* = 0.4776).

*K. pneumoniae* inoculation resulted in acute symptoms associated with a lung infection, as expected as described previously in this paradigm.^11^ A portion of mice were euthanized during the first week post‐injury/infection, the majority between Days 3 and 5 post‐infection, due to excess weight loss ≥20% as per animal ethics guidelines (Table 1). The total mortality rate for vehicle‐treated mice was 5.7% (5.3% for males and 6.3% for females), and for *Kp*‐infected mice it was 22% (17.2% for males and 27.6% for females). The combined insult of TBI and *Kp* infection appeared to increase mortality in male mice; however, this was not the case for female mice. There was no significant difference between mortality in Sham‐*Kp* compared to TBI‐*Kp* mice when analyzed over the first 2 weeks (Figure 1C; Log‐Rank (Mantel−Cox) test, chi‐square test = 0.0063, *p* = 0.9366).

### 3.2 Chronic neurobehavioral outcomes after TBI and *K. pneumoniae* infection

**FIGURE 2:**
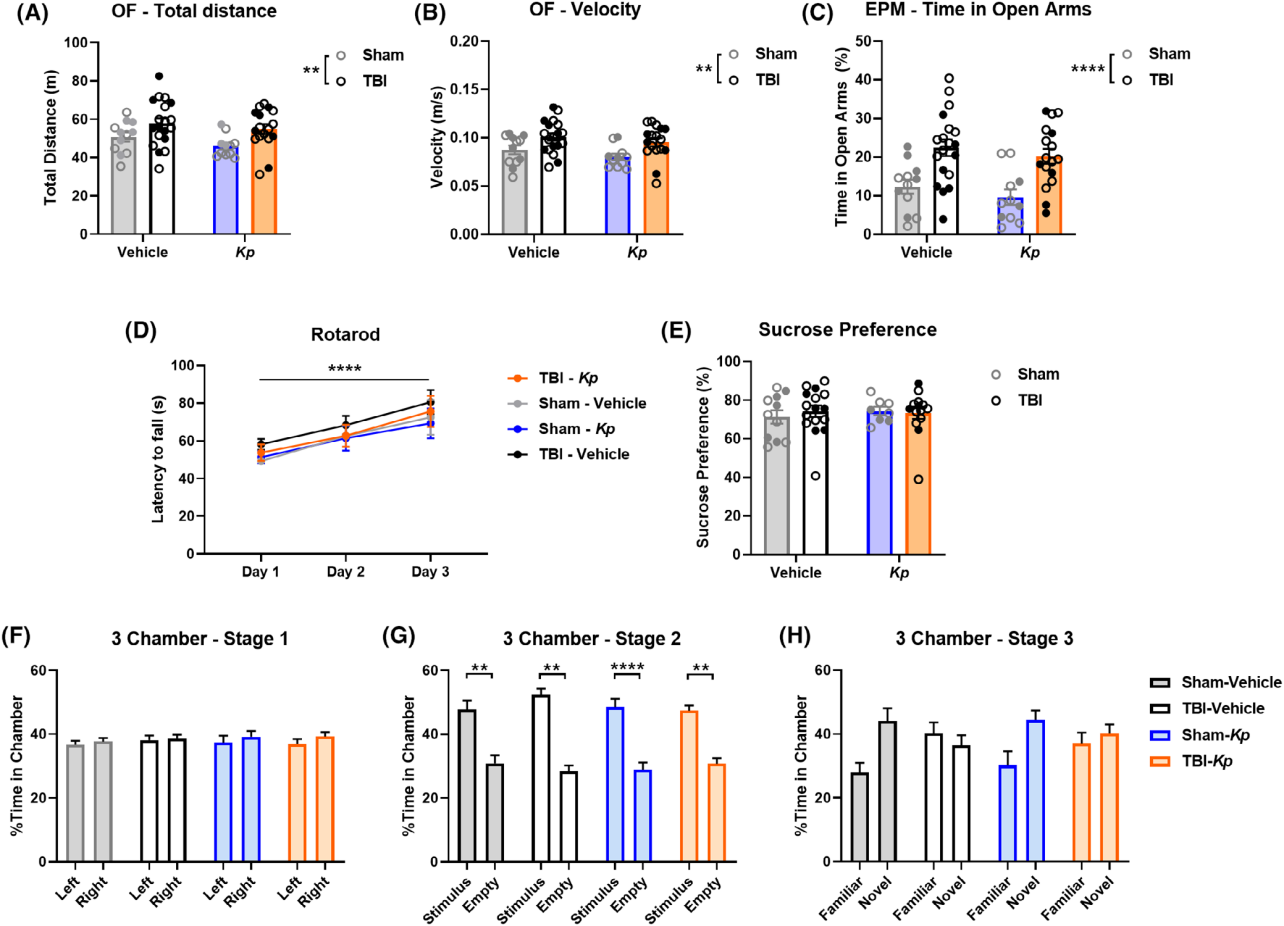
Neurobehavioral outcomes at ~4 months post‐TBI (traumatic brain injury) /*Kp* (*Klebsiella pneumoniae*) infection. (A) Open Field (OF) test, total distance moved. (B) Velocity of movement in the OF test. (C) Time spent in the open arms of the Elevated Plus Maze (EPM). **p < 0.01, ****p < 0.0001, two‐way ANOVA main effect of TBI. (D) Rotarod, latency to fall. (E) Sucrose Preference Test. (F) Three‐Chamber social approach test, Stage 1; (G) Stage 2; and (H) Stage 3. **p < 0.01, ****p < 0.0001, two‐way ANOVA main effect of TBI. In Stage 2, **p < 0.01, ****p < 0.0001 from post hoc comparisons. Open circles = female; closed circles = male.

At ~16 weeks (4 months) post‐TBI/Sham and *K. pneumoniae* infection, all experimental mice underwent a battery of neurobehavioral tests to assess long‐term functional outcomes. Unless stated, no overt sex differences were observed. In the Open Field test, TBI mice showed an increase in total distance traveled compared to the Sham groups (two‐way ANOVA, *F*_1,55_ = 9.06, *p* = 0.0039; Figure 2A), indicating chronic hyperactivity as characterized previously in this model.^10^ Similarly, TBI mice moved with a higher velocity compared to Sham mice (two‐way ANOVA, *F*_1,55_ = 11.28, *p* = 0.0014; Figure 2B). However, *Kp* and vehicle‐treated groups performed similarly, and there were no TBI × infection interactions. Furthermore, no effects of either TBI or infection were observed in terms of the proportion of time spent in the center of the arena (*p* > 0.05).

Next, the Elevated Plus Maze was used as a measure of anxiety‐like behavior (Figure 2C). Here, TBI mice spent significantly more relative time in the open arms of the maze compared to Sham groups (two‐way ANOVA, *F*_1,55_ = 23.79, *p* < 0.0001), indicating a reduction in anxiety‐like behavior chronically after injury as described previously.^10^ Infection did not alter this response.

In the Rotarod test (Figure 2D), although all experimental groups demonstrated improvement in performance over time (increased latency to fall from Trials 1 to 3), no differences between the groups were observed, and there were no interactions between the factors of TBI, infection, or time (three‐way RM (repeated measures) ANOVA, main effect of time *p* < 0.0001). Similarly, all mice showed a comparable preference for sucrose over water in the Sucrose Preference Test (Figure 2E), of ~70%–80%, with no effects of TBI or *Kp* (two‐way ANOVA, *p* > 0.05).

Finally, the Three‐Chamber Social Approach Test was conducted. During habituation in Stage 1 (Figure 2F), as expected, no group differences were observed. In Stage 2 (Figure 2G), a significant main effect of chamber side was detected (three‐way ANOVA, *F*_1,50_ = 87.71, *p* < 0.0001). Tukey's post hoc analyses found that all groups showed a preference for the chamber containing the stimulus animal compared to the empty chamber side, indicating an intact preference for sociability. Finally, in Stage 3 (Figure 2H), a main effect of chamber side was detected (*F*_1,50_ = 6.06, *p* = 0.0173), as well as a chamber side × TBI interaction (*F*_1,50_ = 6.63, *p* = 0.0131). Visually, it appears that Sham‐Vehicle and Sham‐Kp groups spent more time in the chamber containing the novel stimulus mouse compared to the familiar one, whereas both TBI groups spent roughly equivalent time in each chamber. However, Tukey's post hoc analyses failed to detect any specific differences between chamber sides for any of the experimental groups, rendering this stage difficult to interpret.

### 3.3 Chronic seizures after TBI and *K. pneumoniae* infection

Continuous video‐EEG recordings were obtained over an 8–12 day monitoring period per animal at ~5 months post‐TBI/Sham, to evaluate whether early post‐injury *K. pneumoniae* infection altered the development of PTE.

**FIGURE 3:**
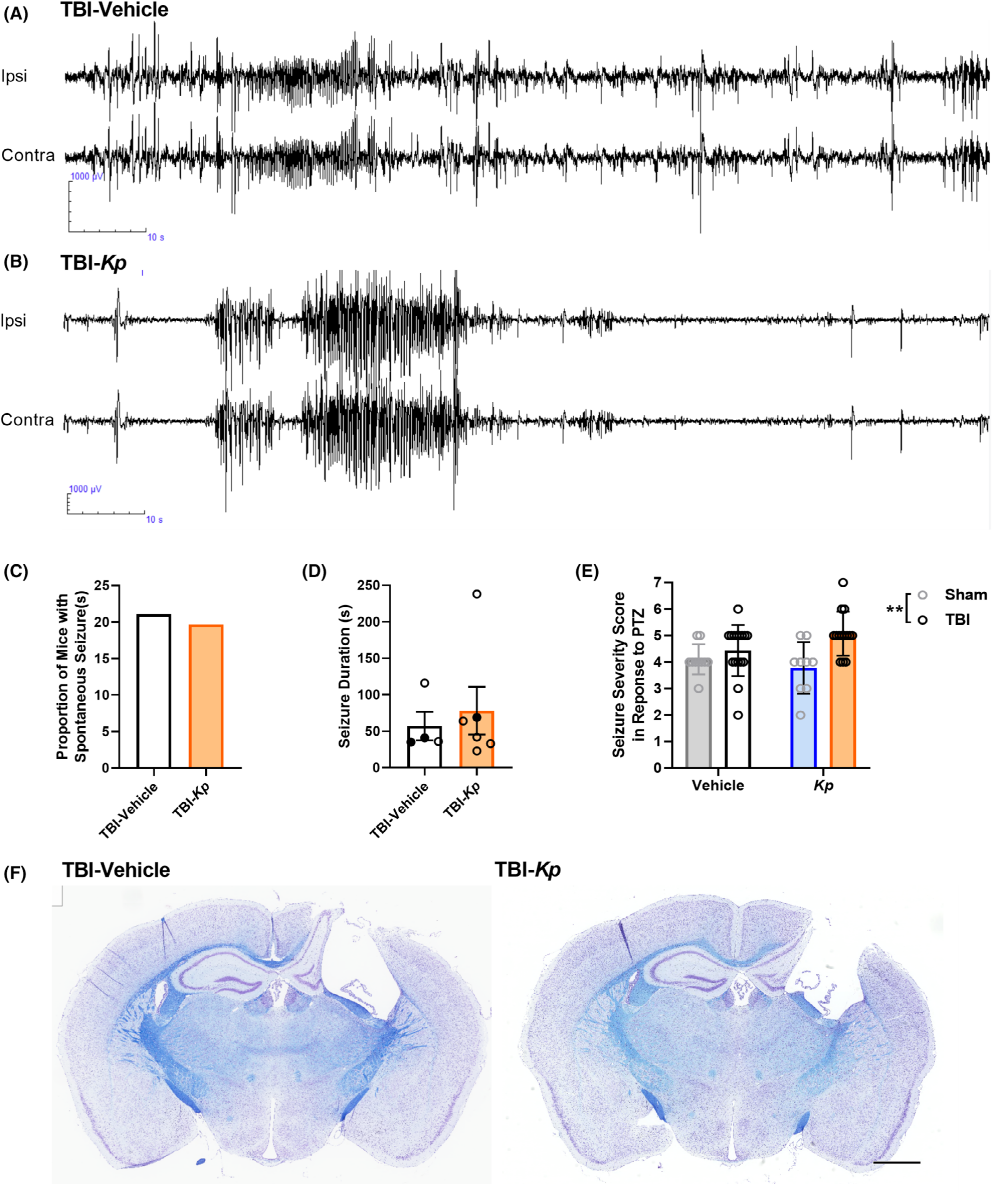
Spontaneous and evoked seizure activity at 5–6 months post‐TBI (traumatic brain injury) /*Kp* (*Klebsiella pneumoniae*) infection. Representative EEG (electroencephalographic) tracings illustrate spontaneous seizure activity in a TBI‐Vehicle (A) and TBI‐*Kp* animal (B) over a 120 s period. Ipsi = electrode positioned ipsilateral to the injury site; contra = contralateral to the injury site. Quantification (C) found a similar proportion of TBI‐Vehicle and TBI‐*Kp* mice exhibiting spontaneous seizures, of a comparable duration (D). Unpaired *t* test, *p* > 0.05. Finally, the evoked response to a PTZ (pentylenetetrazol) challenge was evaluated (E), quantified as the Seizure Severity Score. **p < 0.01, two‐way ANOVA main effect of TBI but no effect of *Kp*. *n* = 12/group for Sham‐Vehicle and Sham‐*Kp*; 19 for TBI‐Vehicle; 16 for TBI‐*Kp*. Coronal brain sections (F) stained with cresyl violet and Luxol Fast Blue illustrate the typical extent of TBI damage observed following the CCI model at this chronic time point, in both TBI‐Vehicle and TBI‐*Kp* mice. Scale bar = 2 mm.

Spontaneous electro‐clinical seizure activity was observed in TBI‐Vehicle and TBI‐*Kp* mice (Figure 3A,B), but none of the sham‐operated mice. All seizures were observed to be generalized in nature, commencing almost simultaneously in both hemispheres (ipsilateral and contralateral to the injury) and were typically Racine Class 3 or 4. Seven animals (four male and three female) exhibited at least one spontaneous seizure, and 40% of seizures occurred during the dark phase. All of the TBI‐Vehicle mice had one seizure each during the recording period. Of the three TBI‐*Kp* mice that had seizures, two had multiple seizures (Table 2). In total, a comparable proportion of Vehicle and Kp‐infected mice were observed to develop PTE: 21.1% of TBI‐Vehicle mice (4/19) and 19.7% of TBI‐*Kp* mice (3/16) (*p* > 0.999, Fisher's exact test; Figure 3C). The average seizure duration was 67 s (median 44 sec; range of 23–238 s) (Figure 3D). Seizure duration was similar across the two experimental groups (*t*_8_ = 0.48, *p* = 0.6421).

**TABLE 2:** Spontaneous seizures observed during chronic video‐EEG monitoring.

ID	Group	Sex	# Seizures	Racine class	Duration (sec)	Light or dark phase
1	TBI‐Vehicle	F	1	4	36	Dark
2	TBI‐Vehicle	M	1	4	41	Dark
3	TBI‐Vehicle	F	1	3	116	Light
4	TBI‐Vehicle	M	1	4	35	Light
5	TBI‐*Kp*	M	1	3	69	Dark
6	TBI‐*Kp*	F	2	4	33	Dark
				4	42	Light
7	TBI‐*Kp*	F	3	3	238	Light
				4	64	Light
				1	23	Light

Upon completion of continuous video‐EEG recording, mice were administered 40 mg/kg, i.p., PTZ and observed for a 15 min period. The behavioral response to PTZ was video‐recorded and then scored by a blinded investigator according to the modified Seizure Severity Score^10^, ^34^ (Figure 3E). Two‐way ANOVA revealed that the average Seizure Severity Score in response to PTZ was higher in TBI compared to Sham mice (main effect of TBI, *F*_1,45_ = 10.41, *p* = 0.0023), whereas *Kp* had no effect (*F*_1,45_ = 0.38, *p* = 0.5406). Latency to the Straub's tail response was also recorded but found to be comparable between groups.

All mice were euthanized after the PTZ challenge and brain tissue collected for histology. A representative image of a TBI‐Vehicle brain is depicted in Figure 3F, illustrating the typical pattern of unilateral cortical and hippocampal damage in the chronic phase as a result of the CCI model.

## 4 DISCUSSION

*K. pneumoniae* is a leading cause of ventilator‐acquired pneumonia in critically‐ill and immunocompromised individuals, and is common in patients with severe TBI.^31^, ^38^, ^39^, ^40^, ^41^ After recently establishing a new mouse model of experimental TBI combined with a pulmonary *K. pneumoniae* infection,^11^ in the current study we sought to investigate if the resolved infection had chronic consequences for TBI outcomes.

In previous pilot studies, we determined 1 × 10^6^ CFU of *K. pneumoniae* ATCC 15380 to be most appropriate for experimental use.^11^ Here we found that this dose induced considerable body weight loss and either spontaneous mortality or substantial weight loss, triggering humane euthanasia for ~20% of infected mice (~15% higher than mortality for vehicle‐treated mice). All mortality occurred within several days of infection, presumably due to pathology associated with the robust innate immune response triggered by *K. pneumoniae* inoculation, as demonstrated previously.^11^ However, the combined insult of TBI plus infection did not exacerbate body weight loss, and there was no additional mortality observed in the TBI‐*Kp* group compared to Sham‐*Kp* mice. Of note, however, sex differences in mortality were observed. While female mice overall appeared to be more susceptible to *K. pneumoniae* infection; the combination of TBI‐*Kp* induced the highest mortality rate in male mice. With mixed reports of sex‐specific responses to *K. pneumoniae* from experimental models,^11^, ^42^, ^43^, ^44^ potential sex‐specific responses to infection warrant further investigation.

Turning to long‐term outcomes, we focused on neurobehavioral function and post‐traumatic epilepsy. Of note, we have demonstrated previously in this model that most of the *K. pneumoniae* bacteria have been cleared from the lungs of surviving mice by 7 days post‐infection, and certainly by a 28‐day time point.^11^ Here we further confirmed that mice had resolved their infection prior to transfer between facilities for behavior testing and video‐EEG, and that no differences were observed between body weight trajectories of any experimental group after the initial 2 weeks (data not shown).

We considered it plausible that prior *K. pneumoniae* infection, despite resolution, may nonetheless have long‐term consequences for neurological function. For example, long‐term deficits in exploratory locomotor behavior were reported in a mouse model of intranasal *K. pneumoniae*–induced pneumosepsis, associated with persistent brain inflammatory gene expression.^45^ We have demonstrated previously that lung infection with *K. pneumoniae* alone induced acute expression of *Tnfα* in the cortex, whereas the combination of TBI plus *K. pneumoniae* resulted in elevated brain gene expression of pro‐inflammatory cytokine *Ccl2* and oxidative stress mediator *Hmox1*.^11^ However, at 4 months post‐injury, we found that previously‐infected *K. pneumoniae* mice were indistinguishable from vehicle‐treated mice across a range of tests for exploratory activity, sensorimotor function, anxiety‐like behavior, social cognition, and pleasure‐seeking behavior. TBI mice displayed chronic hyperactivity and reduced anxiety‐like behavior as expected in this model of CCI.^10^, ^30^

The primary outcome of this study was the evaluation of post‐traumatic seizures, based on recent epidemiological evidence of an association between hospital‐acquired infection and the development of PTE by 2 years post‐injury in patients.^46^ From extended continuous video‐EEG monitoring, we observed spontaneous seizure activity in ~20% of TBI mice, regardless of prior *K. pneumoniae* infection, and their seizures were of a similar nature and duration. In addition, both TBI groups showed a similar response to the PTZ challenge, an indirect measure of seizure susceptibility indicating epileptogenesis. This finding is in line with our previous experimental work using a similar experimental design but LPS as a mimic infectious agent, where LPS failed to have lasting effects on either neurobehavioral or seizure outcomes.^10^

Together, these studies now suggested that either (1) there is not a robust or direct relationship between infection and these chronic outcomes, or (2) the experimental models are still inadequate to replicate the clinical scenario. Of note, although live *K. pneumoniae* in this study was administered into the lungs (a clinically‐relevant route), it was delivered as a single bolus, which does not represent how a bacterial load expands over a time course in infected patients.^14^, ^47^, ^48^ This may account for some of the mortality we observed, but may also influence the resulting immune response including brain–lung–immune interactions. Furthermore, inherent differences between human and rodent immunity may render the mouse an insufficient model system for investigating the complex scenario of a critically‐ill patient with TBI and infection.^14^ It is also possible that inadvertent selection bias due to higher mortality in TBI‐*Kp* mice may have influenced comparison with TBI‐Vehicle mice. In other words, mice that succumbed to *Kp* infection may have been the most severe animals, leaving a survivor cohort that were more “mild” in severity. Future experiments in which TBI of differing severities is combined with *K. pneumoniae* infection are needed to understand whether TBI severity influences mortality in response to infection.

Several strengths and limitations of the current study are worth noting. The study was carefully designed to dissect out specific effects of the combined insult of TBI plus *K. pneumoniae* compared to either TBI or infection alone, and is one of few studies to date to consider the long‐term consequences of such a paradigm. Detailed seizure monitoring via continuous video‐EEG is the gold‐standard measure of PTE, and we supplemented this with evaluation of PTZ‐evoked seizure responses as an additional surrogate indicator. The inclusion of both male and female mice allowed for consideration of typical biological variability as well as exploration of potential sex differences in responses to injury and infection. The lack of any sex differences chronically was not unexpected given that acutely, sexual differentiation was observed only in relation to *K. pneumoniae* infection, which did not have persistent effects on behavior or seizure susceptibility independent of the observed TBI effects.

Conversely, the study was limited by lack of longitudinal analysis of seizure development, which could provide additional insight into seizure onset and progression over time. Use of a single infection model, time of administration relative to injury, and host strain/species also limit the generalizability of the findings to other types of infections or pathogens. Adjustments to the infection model, use of alternative pathogens, or use of immunocompromised host animals may allow for more chronic infections to be investigated in the context of neurotrauma. Furthermore, we did not examine early post‐injury seizures, which may be influenced by additional immune challenges such as an infection, and have themselves been implicated as a risk factor for PTE.^46^ Finally, although this study focused on behavioral and seizure outcomes, we did not delve into the biological or molecular mechanisms that underlie these presentations. Future studies may benefit from the complementary inclusion of detailed histological or molecular analyses.

We also acknowledge the technical challenges inherent in preclinical modeling of PTE. The incidence of spontaneous chronic seizures observed herein falls within the wide range of reported incidence of PTE from human populations.^16^, ^17^, ^18^, ^29^, ^49^, ^50^, ^51^ The development of late seizures in only a subset of animals poses both an advantage (allowing for the examination of experimental manipulations that are hypothesized to increase seizure incidence) and a challenge (obtaining sufficient power). Future studies of this nature may consider additional tests to provide greater insight into seizure threshold changes (e.g., use of electroconvulsive seizure thresholds), and would benefit from international collaborative efforts to provide increased sample size and cross‐laboratory validation.

## 5 CONCLUSION

In conclusion, this study provides important insights into the potential long‐term effects of *K. pneumoniae* infection following moderate‐to‐severe TBI. We found that a non‐trivial *K. pneumoniae* lung infection, associated with ~20% mortality, did not affect the chronic TBI‐induced neurobehavioral deficits or PTE for the survivors. These findings suggest that although *K. pneumoniae* can worsen acute health parameters, its impact on long‐term neurological outcomes post‐TBI is limited once the infection has been resolved. Further research is needed to further explore potential sex‐specific responses to infection, and consider how the timing of infection relative to injury may influence the host's response. Overall, this work contributes to our growing understanding of how infections influence brain injury outcomes. Given the high incidence of both infections and seizures in TBI patients, and a growing concern over multidrug‐resistant pathogens, such research is vital to the medical management of infections in patients with TBI and support of optimal long‐term outcomes.

## AUTHOR CONTRIBUTIONS

Project conceptualization: B.D.S., T.O.B., and J.L. Data access and analysis: B.D.S., A.S., S.S.R., L.C., E.C., K.C., J.W., and P.M.C.E. Manuscript draft: B.D.S. Manuscript edits: All authors. We confirm that this manuscript is consistent with the Journal's and publisher's position on issues involved in ethical publication.

## FUNDING INFORMATION

This project was supported by an Epilepsy Research Program Idea Development Award (#W81XWH‐19‐ERP‐IDA) from the U.S. Department of Defense, awarded to B.D.S., J.L., and T.O.B. B.D.S. was also supported by a Veski Near‐Miss Grant. T.O.B. is supported by a National Health and Medical Research Council of Australia (NHMRC) Investigator Grant (APP1176426). J.L. is supported by an NHMRC Principal Research Fellowship (APP1157909). P.M.C.E. is supported by the NHMRC (APP1087172 and APP2013629), a Monash Future Leader Fellowship (FLPF24‐0237761657), a Medical Research Future Fund (MRFF), Australian Government, Department of Health, Disability and Ageing Stem Cell Therapy Missions Grant (MRF1201781), and the U.S. Department of Defense USA Epilepsy Research Program (DoD ERP IDA, grant # EP200022, DoD ERPA RPA, grant# EP220067).

## CONFLICT OF INTEREST STATEMENT

The authors do not have any conflicts of interest to declare. The funders had no role in the design of the study; in the collection, analyses, or interpretation of data; in the writing of the manuscript; or in the decision to publish the results. We confirm that we have read the Journal's position on issues involved in ethical publication and affirm that this report is consistent with those guidelines.

## ETHICS APPROVAL

All animal experiments were conducted following approval from the local Alfred Research Alliance Animal Ethics Committee (#P8032) as well as the Animal Care and Use Review Office (ACURO) from the Office of Research Protections, U.S. Department of Defense, and carried out in accordance with these approved standards as well as the Australian Code for the Care and Use of Laboratory Animals as stipulated by the National Health and Medical Research Council of Australia (NHMRC).

## Supporting information


Data S1.

## Data Availability

The data that support the findings of this study are available from the corresponding author upon reasonable request.
