# Supplementary material for: Chronic behavioral and seizure outcomes following experimental traumatic brain injury and comorbid Klebsiella pneumoniae lung infection in mice
Source: Epilepsia. 2025 Jul 15;66(11):4513–24. doi: 10.1111/epi.18551 (PMC12661288; doi:10.1111/epi.18551)
Supplement: Supplementary file 1 — Data S1. [file EPI-66-4513-s001.docx]

**SUPPLEMENTARY METHODS**

**Chronic Behavioral and Seizure Outcomes following Experimental Traumatic Brain Injury and Comorbid *Klebsiella pneumoniae* Lung Infection in Mice**

Sarah S. J. Rewell^1^, Ali Shad^1, 2^, Lingjun Chen^1^, Erskine Chu^1^, Jiping Wang^3^, Ke Chen^3^, Terence J. O’Brien^1, 2, 4^, Jian Li^3^, Pablo M. Casillas Espinosa^1,2^, Bridgette D. Semple^1^ *

The initial experiments (TBI surgeries and *K. pneumoniae* inoculations) were carried out in QC2 (microbiological containment) facilities at the Monash Research Animal Precinct in Clayton, Australia, before mice were transferred to the Precinct Animal Centre at the Alfred Hospital in Melbourne, for a 4-week quarantine period before behavior testing and video-EEG. The mice were housed in groups of same-sex littermates (2-6 per cage; with mixed experimental conditions per cage) in Optimice® individually-ventilated cages, maintained on a 12-hour light/dark cycle with continuous access to food and water.

Controlled cortical impact (CCI) model of TBI

Moderate-to-severe experimental traumatic brain injury (TBI) was induced in 10-12 week old mice using the controlled cortical impact (CCI) model, as previously described^30^ (see Supplementary Methods). Briefly, anesthesia was initiated with 4% isoflurane in oxygen and maintained at 1.5% via a nose cone. Prior to surgery, all animals were given buprenorphine (0.05 mg/kg in saline; subcutaneously in the flank) and bupivacaine (1 mg/kg in saline; subcutaneously in the scalp) for pain relief, and 0.5 mL of 0.9% saline was administered at the end of the procedure for hydration. The mice were stabilized in a stereotaxic frame, and a 3.5 mm craniotomy was performed over the exposed left parietal bone. An electronic controlled cortical impactor device (eCCE-6.3; Custom Design and Fabrication Inc., Sandston, VA) was used to deliver an impact with a 3 mm rounded tip, at a speed of 4.5 m/s, to a depth of 1.7 mm for 150 ms. Sham animals underwent the same surgical procedure without the impact. After the CCI or sham surgery, the skin incision was sutured and an antiseptic solution applied. The animals were then allowed to recover in individual cages on a heat mat before being returned to their home cage.

Facility Transfer

The initial experiments (TBI surgeries and *Kp* inoculations) were carried out in QC2 facilities at the Monash Research Animal Precinct in Clayton, Australia, before mice were transferred to the Precinct Animal Centre at the Alfred Hospital in Melbourne for behavior testing and video-EEG monitoring. This experimental design was required due to the specialized facilities required for these procedures. Prior to leaving the Monash Clayton facility, for each of 4 cohorts of mixed-experimental groups, a health screen was performed at 6 weeks post-inoculation by sacrificing and testing a Swiss strain male mouse that was housed alongside the experimental mice and exposed to their bedding weekly. An additional health check was performed at the end of the four-week quarantine period upon arrival at the Alfred Hospital site. This involved the sampling of sera collected from 2-4 experimental animals that had previously had *K. pneumoniae* infection, via submandibular bleed performed by the animal facility technicians. Health screening was performed by Cerberus Sciences (Scoresby, VIC, Australia) via a standard panel. All cohorts passed the health screening. Once through the quarantine period, mice were transferred to the holding rooms in the Department of Neuroscience at Monash University, Alfred Hospital, and habituated for at least one week before behavior testing.

Behavior Testing

At approximately 16 weeks post-injury, a comprehensive battery of neurobehavioral tests was conducted to assess the chronic consequences of TBI and *K. pneumoniae*. Firstly, mice underwent an Open Field (OF) test, in a square arena for 10 min duration, to evaluate general locomotor activity, exploratory behavior and anxiety-like behavior. Anxiety-like behavior was additionally measured using the Elevated Plus Maze (EPM) during a 10 min period. In both tests, TopScan software was used to track activity and the time spent in the center versus periphery or open arms compared to the closed arms of the maze.^30^

Gross sensorimotor performance was evaluated using the accelerating rotarod test over three consecutive days. Each day, mice completed three trials with a 30-minute rest period between trials. The rotarod device accelerated from 4 to 40 rpm over a 5-minute period, which was the maximum duration of the test. The average latency to fall from the rotarod was calculated for each mouse per day.^30^

Social approach and social novelty preferences were assessed using the three-chamber test, which involved three consecutive 10-minute sessions conducted with a custom-built Perspex apparatus. The test proceeded through three stages: first, a habituation period (stage 1); then, the introduction of a same-sex stimulus mouse into one of the outer chambers (stage 2); and finally, the placement of a second, novel same-sex stimulus mouse into the opposite outer chamber (stage 3). TopScan software was used to monitor the amount of time the experimental mouse spent in each outer chamber. In stage 2, a preference for the chamber with the stimulus mouse over the empty chamber indicated social interest, while in stage 3, a preference for the novel mouse over the familiar mouse demonstrated social recognition or memory.^34, 35^ 2 mice from the TBI-Vehicle group were unable to be analyzed from this test due to aberrant video tracking.

Finally, the sucrose preference test was employed to identify potential depressive-like anhedonia in mice. Over a 5-day period, mice had access to two drinking bottles: one with filtered water and the other with a 1% sucrose solution. The positions of the bottles were switched halfway through the experiment. On the first and last day of the test, the volumes of liquid consumed from each bottle were measured, and a sucrose preference ratio was calculated by dividing the volume of sucrose solution consumed by the total volume of liquid consumed.^10^

Postmortem Lesion Assessment

Mice were humanely euthanized via intraperitoneal injection of 160 mg/kg sodium pentobarbitone (Lethabarb®, Virbac, Australia), followed by transcardial perfusion with 4% paraformaldehyde (PFA) at a rate of 2 mL/min. The extracted brains were post-fixed overnight in 4% PFA, then transferred to 70% ethanol and sent to the Monash Histology Platform for paraffin processing and embedding (Monash University, Clayton, Australia). Seven μm coronal brain sections were sectioned and stained with cresyl violet and Luxol Fast Blue, as described previously,^11, 30^ to illustrate the extent of pathology.

4310
